# Experimental Study of the Effect of High Service Temperature on the Flexural Performance of Near-Surface Mounted (NSM) Carbon Fiber-Reinforced Polymer (CFRP)-Strengthened Concrete Beams

**DOI:** 10.3390/polym13060920

**Published:** 2021-03-17

**Authors:** Younes Jahani, Marta Baena, Javier Gómez, Cristina Barris, Lluís Torres

**Affiliations:** AMADE, Polytechnic School, University of Girona, 17003 Girona, Spain; marta.baena@udg.edu (M.B.); javier.gomez@udg.edu (J.G.); cristina.barris@udg.edu (C.B.); lluis.torres@udg.edu (L.T.)

**Keywords:** NSM strengthening, carbon fiber reinforced polymer, epoxy adhesive, temperature, experimental, shrinkage

## Abstract

This paper presents a study of the effect of high service temperature (near or beyond glass transition temperature (*T*_g_) of structural epoxy adhesive) on the behavior of near-surface mounted (NSM) carbon fiber-reinforced polymer (CFRP)-strengthened reinforced concrete (RC) beams. The study includes experimental work as well as analytical and numerical analysis. To this end, fourteen beams have been tested up to failure in two different series. In series 1, specimens with three different CFRP areas have been tested at two different temperatures (i.e., 20 and 40 °C). In series 2, and with the aim of evaluating the effect of higher temperatures, only one CFRP area was tested under four different temperatures (i.e., 20, 60, 70, and 85 °C). Experimental results are evaluated in terms of load–deflections, failure modes, and bond performance. Furthermore, the experimental load–deflection curves are satisfactorily compared to both analytical predictions and finite element (FE) numerical simulations. In both cases, shrinkage and temperature effects on the short-term response of flexural elements have been accounted for. No significant reduction in stiffness and ultimate load was observed for specimens being tested up to 60 °C (in the range of epoxy *T*_g_), showing FRP rupture failure in all of them. For specimens under 70 and 85 °C, the failure mode changed from FRP rupture to FRP end debonding and concrete crushing, respectively.

## 1. Introduction

In the past decades, fiber-reinforced polymer (FRP) materials have been produced in different configurations and have been widely used for different purposes, such as the strengthening of reinforced concrete (RC) structures. Currently, externally bonded reinforcement (EBR) and near-surface mounted (NSM) are the two most used strengthening techniques in civil structures. In the EBR technique, the FRP is bonded to the previously prepared concrete surface, usually with an epoxy resin, while in the NSM technique, a groove is cut in the concrete cover and the FRP bar or strip is inserted and bonded using groove filler, which is typically an epoxy adhesive or a cement grout. The improvement of the bond performance, along with higher protection against potential aggressive environmental exposure and vandalism actions, are some of the advantages of the NSM technique [1,2,3].

The performance of the NSM strengthening system relies on the bond capacity of the joint between concrete and FRP material, which in turn depends on the properties of the adhesive (usually epoxy resin), among other parameters. Typically, epoxy adhesives can be affected by temperature, as near or beyond the glass transition temperature (*T*_g_), their mechanical properties may change [4,5,6,7]. This may lead to a decrease of its performance and premature debonding and therefore to a not complete exploitation of the strengthening system. In this sense, some limitations on the working temperature of these strengthening systems exist. According to fib Bulletin 90 [8], to avoid any considerable change in the adhesive properties in the service condition, the maximum temperature should be 20 °C less than *T*_g_. In this same line, Michels et al. [9] summarized the various design codes provisions to define the service temperature in FRP strengthened RC structures to be 10 to 20 °C less than *T*_g_, whilst Klamer et al. [10] suggested the service temperature to be limited to 10 °C less than *T*_g_. Furthermore, Ferrier et al. [11] limited the service temperature to be 15 °C less than *T*_g_ for avoiding temperature effects in the creep of epoxies with lower range of *T*_g_ (*T*_g_ < 55 °C). Moreover, other studies [12,13] limited the maximum temperature to 10 °C less than *T_g_* to avoid premature debonding of FRP sheets from concrete surface. These limitations are related to the EBR technique, while for the NSM technique, less information is available, and no specific limitation has been stated.

Focusing on the existing experimental work, the flexural behavior of NSM strengthened concrete beams under room temperature have been widely studied in the literature [14,15,16,17,18,19,20], while the structural performance of this technique under elevated temperature and high service temperature is still an open topic that needs detailed research. The fire resistance of FRP-strengthened RC flexural elements has been analyzed in different studies [21,22,23,24,25,26,27,28,29,30]. Results from these works showed that the efficiency of the NSM method was better than EBR technique, and the thicker layers of the insulation system helped significantly reduce the temperature in the concrete and the adhesive. As a result, failure in the interface between laminate and concrete was postponed and, therefore, the durability of the system increased. A deep state-of-the-art review on the performance of FRP-strengthened RC elements exposed to fire conditions can be found in [31].

Compared to fire resistance, the performance of FRP-strengthened RC flexural elements exposed to high service temperature has been hardly investigated. The behavior of four different EBR strengthened RC beams under three different temperatures (i.e., 20, 50, and 70 °C), was studied and analyzed in [32]. Results showed that the failure load of the specimens was not significantly affected by temperature. However, a change in the failure interface took place for specimens with shorter anchorage length exposed to higher temperature (70 °C). Similar results were obtained in [33], who applied temperatures in the range of 20 to 80 °C and concluded that the load capacity of EBR-strengthened beams subjected up to 62 °C was slightly decreased, whilst the application of larger temperatures (from 70 °C on) resulted in a reduction in load capacity of approximately 20% of its nominal capacity at room temperature. Moreover, the application of larger temperatures made the failure mode change from cohesive in concrete to adhesive at the concrete–epoxy interface. Moving from beams to slabs, Silva et al. [34] studied the flexural performance of concrete slabs strengthened with the NSM technique under service temperature (up to 80 °C). According to this work, the maximum ultimate load capacity was observed in a slab under 40 °C, which was attributed to possible post-curing in epoxy adhesive, and the increase in the temperature up to 80 °C was followed by a decrease of the ultimate capacity of the slab of about 12%. In addition, the specimens subjected to 80 °C failed by cohesive failure at the epoxy, while in the rest of the specimens, the failure mode was concrete crushing. Focusing on EBR adhesive double lab joints with a wet lay-up system, Ferrier et al. [35] investigated the effect of temperature (ranging from −40 to 120 °C). According to the experimental results, the increase of temperature was followed by an increase of the slip between joint elements that made the specimens failed at lower loads.

In addition to experimental work, finite element methods have been also widely used to simulate the FRP-strengthened beams and columns under different loading and environmental conditions [36,37,38,39,40,41,42,43]. In these studies, different types of FRP material (i.e., carbon, glass, and basalt) have been considered as NSM or EBR reinforcement, and both the flexural and shear performance of the strengthened members have been validated. It should be noted that these studies correspond to specimens under room and fire conditions.

According to the literature, it is seen that there are a limited number of studies related to the flexural performance of NSM FRP-strengthened RC beams under high service temperature, which justifies the need for further research in this field. The present work aims at studying the effect of relatively high service temperature (near and beyond *T*_g_) on the flexural performance of NSM carbon FRP (CFRP)-strengthened RC beams when different strengthening areas of CFRP are used. For this purpose, an experimental program has been carried out, and results are analyzed in terms of flexural behavior, failure modes, and bond performance along the FRP laminate. Additionally, experimental results are also compared to analytical predictions and numerical simulations with the finite element method. The influence of shrinkage and temperature on the short-term response of the specimens was included in both analytical calculations and numerical predictions.

## 2. Experimental Program

### 2.1. Experimental Test Setup

**Figure 1:**
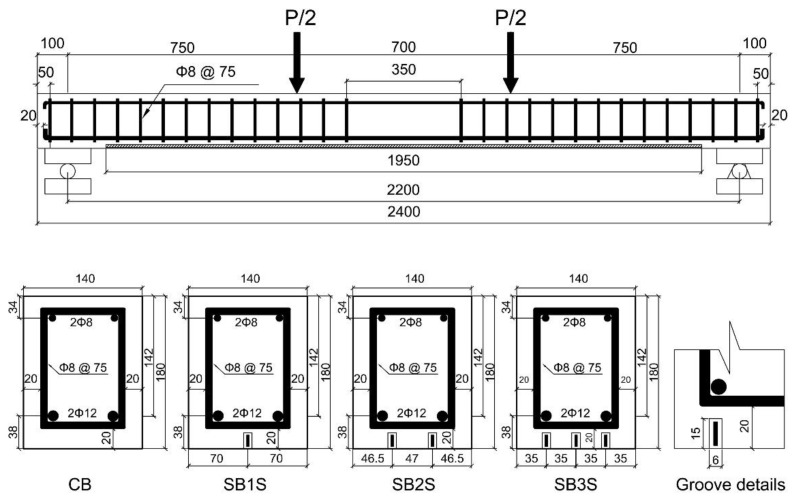
Beam details (dimensions in mm).

The experimental program included fourteen specimens divided into two series (Table 1). Series 1 included eight beams, which were distributed as follows: two control beams (exposed to 20 and 40 °C) and six NSM CFRP-strengthened beams (exposed to 20 and 40 °C) with three different areas of CFRP. In series 2, unlike series 1, only one CFRP-strengthening ratio was used, and four different temperatures were applied (20, 60, 70, and 85 °C). The beams were tested under a four-point bending configuration (Figure 1). The beams had a rectangular cross-section of 140 × 180 mm and a total length of 2400 mm. The clear span was 2200 mm and the shear span was 750 mm, thus leading to a flexural span of 700 mm. Two ribbed steel bars with a diameter of 12 mm were utilized as longitudinal reinforcement on the tension side, while two ribbed steel bars with the diameter of 8 mm were used on the compression side. The shear span and some portion of the flexural span were reinforced with ϕ8 mm steel stirrups every 75 mm to avoid shear failure. All beams had a 5 mm wide and 15 mm deep notch at midspan (to act as a crack initiator at this specific position).

The same CFRP strip, having a thickness of 1.4 mm and a width of 10 mm, was used in all the strengthened beams, but different configurations of the strengthening system were considered (see Figure 1). The bonded length of the CFRP strips was 1950 mm for all specimens. Grooves of size 6 × 15 mm were cut using a sawing machine. After cutting them, the grooves were completely cleaned with air pressure to make sure there was no dust inside the grooves.

The specimens were designated as X-Y-Z, where X denotes the type of beam (CB for control beams with no strengthening system, SB1S for beams strengthened with one CFRP strip, SB2S for beams strengthened with two CFRP strips, and SB3S for beams strengthened with three CFRP strips), Y indicates the series of specimens, and Z is the temperature applied to the beam (where R refers to room temperature equal to 20 °C). For example, SB1S-1-R refers to the strengthened beam with one strip in series 1 of specimens at 20 °C. Details of the specimens are shown in Table 1.

**Figure 2:**
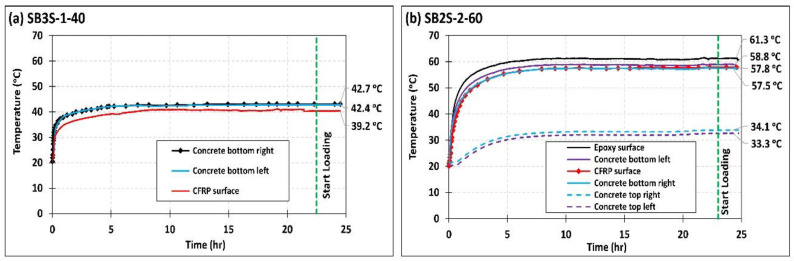
Evolution of temperature with time at different locations of specimens (**a**) SB3S-1-40 and (**b**) SB2S-2-60 (before and during loading).

Beams to be tested at high temperature were submitted to a heating process before instantaneous testing. The heating was applied on the tension face of the beam by using silicone rubber fiberglass-reinforced heating blankets. An insulating support was used to hold the heating blankets in contact with the tension face of the beam. A proportional integral derivative (PID) controller was utilized for the heating process, and Type-T thermocouples, located between the heating blankets and the concrete surface, were used as temperature controlling sensors. The evolution of temperature with time at different locations is shown in Figure 2. Tests started after the stabilization of temperature (see Figure 2), which remained constant along the test. Temperature strain gauges located at different points of the beam section allowed the registering of temperature along both the heating process and flexural test.

In all the cases, a hydraulic jack applied the load onto the specimens through a spreader beam. The load was applied under displacement control mode at a rate of 0.6 mm/min.

### 2.2. Instrumentation

**Figure 3:**
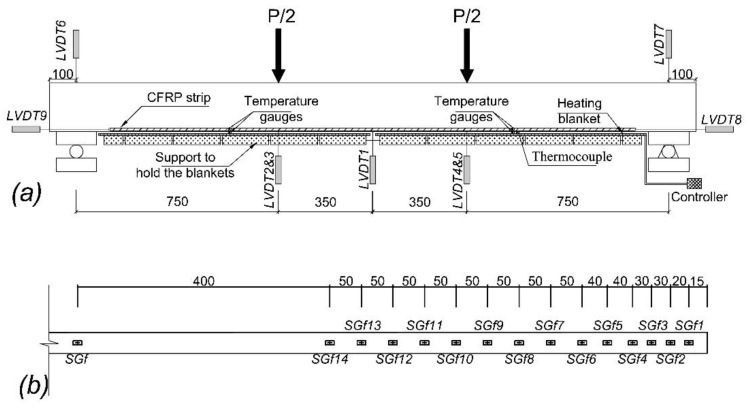
Test configuration: (**a**) position of linear vertical displacement transducers (LVDTs), temperature gauges, thermocouples, and heating system; (**b**) strain gauges along the carbon fiber-reinforced polymer (CFRP) laminate in specimens SB1S-1-R and SB1S-1-40 (dimensions in mm).

Five linear vertical displacement transducers (LVDTs) were used to record the vertical displacements, and two other LVDTs were utilized to measure the settlement of the supports. Due to the use of heating blankets in the soffit of the beam, two LVDTs were installed to the side faces of the beam under each loading point. Furthermore, end slips of the NSM CFRP strip were measured with two horizontal LVDTs (see Figure 3a). The measurements from horizontal LVDTs were almost negligible in all specimens.

A strain gauge was installed at the midspan section of specimens on FRP surface (SGf) to assess strain variation with load. Furthermore, to evaluate the bond behavior between the CFRP strip and concrete, 14 additional strain gauges (SGf1 to SGf14) were installed along the strengthening strip for SB1S-1-R and SB1S-1-40 specimens (see Figure 3b). To avoid reducing the bonded surface, the strain gauges were installed in both sides of the strip (i.e., alternate in front and back of the strip).

**Figure 4:**
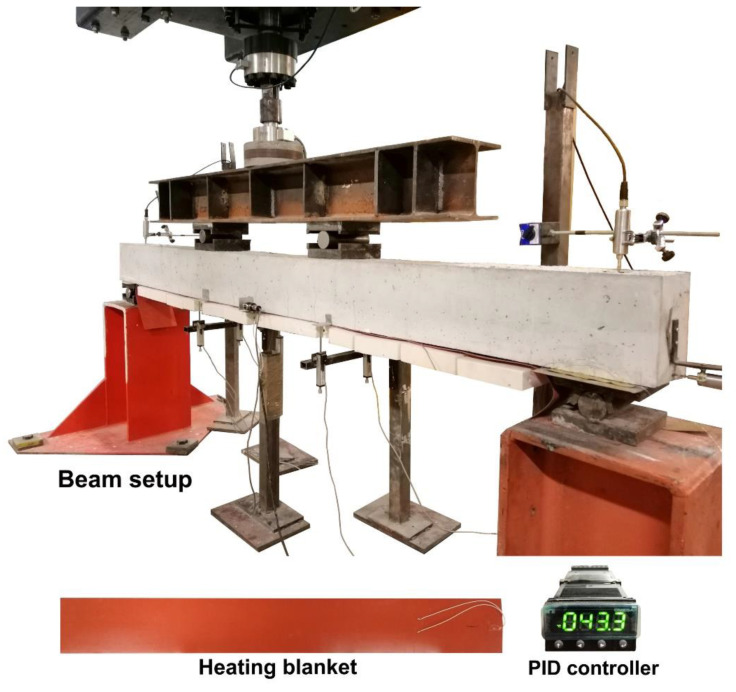
General overview of test scene and elements of the heating system.

Moreover, to record the temperature variation, temperature gauges (thermocouples) were installed in specimens under temperature at the surface of the concrete at the top and soffit of the beam and inside the groove. The temperature variations were recorded during the heating process as well as during the flexural tests. The position of the thermocouples is shown in Figure 3a. A general overview of test scene, along with the elements of the heating system (PID controller and heating blanket) is shown in Figure 4.

### 2.3. Materials

The specimens were cast in lab conditions with conventionally vibrating procedure. Two different concrete batches were used. For concrete of series 1, the cement type was I-42.5R, with a content of 390 kg/m^3^, the maximum aggregate size was 12 mm, and the water/cement relationship was 0.46. For concrete of series 2, the cement type was I-42.5R, with a content of 333 kg/m^3^, the maximum aggregate size was 10 mm, and the water/cement relationship was 0.48. In both series, a viscosity modifier and underwater admixture were used to improve workability. The experimental compressive strength, tensile strength, and modulus of elasticity of concrete were determined from three cylinder tests (300 mm high and 150 mm diameter) according to UNE-EN 12390-3:2003 [44], UNE-EN 12390-6:2010 [45], and ASTM C469-87 [46] standards, respectively. According to the test results, for series 1 of specimens, an average compressive strength of 31.8 MPa (CoV = 6.6%), an average tensile strength of 4.2 MPa (CoV = 2.3%), and an average modulus of elasticity of 31.5 GPa (CoV = 7.8%) were obtained. Furthermore, for series 2 of specimens, the compressive strength, tensile strength, and modulus of elasticity were 40.8 MPa (CoV = 2.8%), 5.2 MPa (CoV = 0.5%), and 29.4 GPa (CoV = 0.8%), respectively.

The mechanical properties of steel bars were obtained from tension tests based on UNE-EN ISO 15630-1 [47]. The yielding stress and the modulus of elasticity were 573.2 MPa (CoV = 1.1%) and 200.8 GPa (CoV = 0.7%), respectively.

The CFRP laminates used in the experimental work consisted of unidirectional carbon fibers (with a volume content fiber higher than 68%) held together by an epoxy vinyl ester resin matrix [48]. The mechanical properties were obtained according to ISO 527-5:2009 [49] recommendations. An ultimate tensile strength of 2251.4 MPa (CoV = 3.2%), an ultimate tensile strain of 0.0133 (CoV = 7.2%), and modulus of elasticity of 169.5 GPa (CoV = 6.3%) were obtained.

A two-component epoxy adhesive was used to bond the CFRP strip to a concrete groove. According to the manufacturer’s product guide specification [50], the components A and B should be mixed at a ratio of 2:1 by weight. In addition, the proposed curing time was 7 days. However, in this study, the specimens were tested after 12 days of epoxy curing at room temperature.

Mechanical properties of the epoxy adhesive were assessed according to ISO-527-1 [51]. A modulus of elasticity of 7.1 GPa (CoV = 7.8%) and tensile strength of 30.4 MPa (CoV = 4.3%) were obtained.

**Figure 5:**
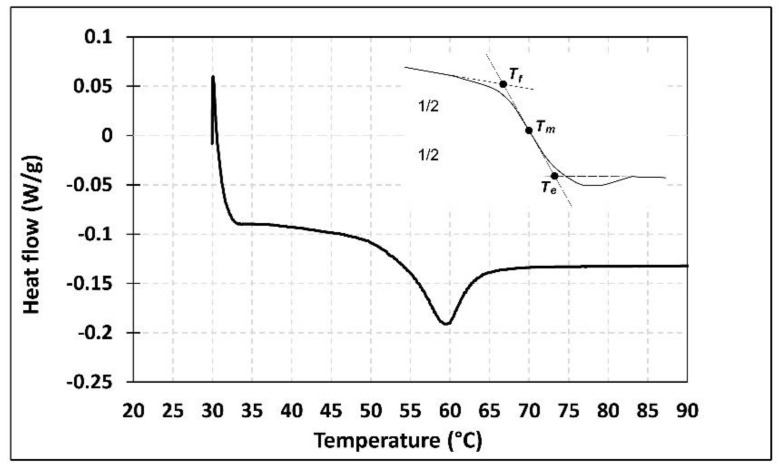
Differential scanning calorimetry (DSC) test results.

**Figure 6:**
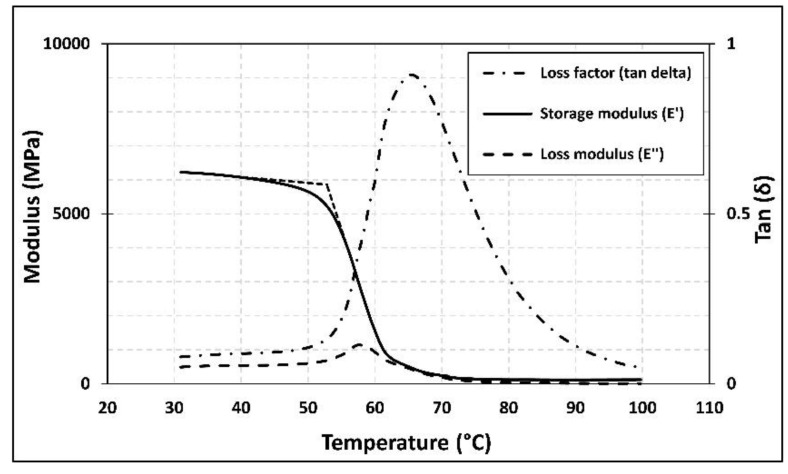
Dynamic mechanical analysis (DMA) test results.

As mentioned previously, the aim of this study is to evaluate the effect of high service temperature on the flexural behavior of NSM CFRP-strengthened beams. Therefore, characterization of the glass transition temperature (*T*_g_) of the epoxy adhesive is of interest. According to the literature, there are two different well-known methods to obtain *T_g_* of the adhesive: differential scanning calorimetry (DSC) [52] and dynamic mechanical analysis (DMA) [53]. The DSC technique provides information about changes in physical, chemical, and heat capacity of the adhesive. In this experimental program, isothermal DSC tests were carried out using the DSC Q2000. A heating rate of 10 °C/min was applied using nitrogen as the purge gas at 50 mL/min. The temperature range was between 25 and 80 °C. To determine *T*_g_, three temperatures were measured: the extrapolated onset temperature (*T*_f_), the mid-point temperature (*T*_m_), and the extrapolated end temperature (*T*_e_). The DMA test is an alternative method to determining the *T*_g_ and viscoelastic properties of polymeric materials. In this study, a METTLER TOLEDO DMA/SDTA861e analyzer with a 3-point bending test configuration and 45 mm between supports was utilized. The specimens were subjected to a heating rate of 2 °C/min within a temperature range of 30 to 100 °C. A 5 µm, constant displacement amplitude was applied at a frequency of 1 Hz. *T*_g_ was obtained by analyzing the storage modulus (*E′*), the loss modulus (*E″*), and the loss factor (*tanδ*) as functions of temperature. In the present work, both methods (DSC and DMA) were utilized to determine the *T*_g_ after 12 days of curing time at room temperature. According to the test results, presented in Table 2, *T*_g_ cannot be considered as a unique temperature, but rather, it should be considered as a range of temperatures, as stated elsewhere [9]. The results of DSC and DMA tests are plotted in Figure 5 and Figure 6, respectively. According to these results, *T*_g_ was in the range of 53.9–65.3 °C.

## 3. Results and Discussion

In this section, experimental results are presented and discussed in terms of load–deflection curve, failure mode, and strain distribution along the CFRP strips. Analytical predictions for the load–deflection relationship are also presented.

In the analytical work, equilibrium of forces and moments is applied, where the following assumptions have been considered: (i) strain compatibility; (ii) Bernoulli’s hypothesis; (iii) perfect bond, and (iv) the parabola–rectangle stress–strain curve defined in Eurocode 2 [20]. Based on this, theoretical moment–curvature is determined for uncracked and cracked sections and, finally, the moment–curvature relationship is derived as the interpolation between cracked and uncracked sections, according to CEB-FIP Model Code 1990 [54]. Afterwards, an analytical load–deflection curve is obtained from an integration of curvatures. It should be noted that shrinkage previous to loading has been found to affect the load–deflection behavior of RC members [55,56,57,58]. This influence has also been experienced in the NSM FRP-strengthened beams included in this work. Therefore, a systematic procedure is applied to account for this effect in the analytical predictions for their load–deflection relationship.

### 3.1. Shrinkage Effects on Instantaneous Deflection of Concrete Beams

Concrete shrinkage is a reduction in its volume due to moisture loss. This reduction starts after casting the concrete and depends on the water/cement ratio, ambient humidity, shape of aggregates, mixture properties, curing method and temperature, and geometry of specimens, among other parameters. In plain concrete without reinforcement, this shortening would happen without any restrictions. However, in the case of reinforced concrete, the presence of the embedded reinforcement acts as an impediment to free shrinkage. As a result of the restrain provided by the reinforcement, compressive and tensile loads appear in the reinforcement and the concrete, respectively, therefore causing unsightly cracks in concrete. Additionally to these premature cracks, in those cases where non-uniform distribution of reinforcement in section depth exists, an additional curvature can be observed [58].

Shrinkage is an important parameter in predicting the long-term deflection of RC flexural members, although depending on the amount of shrinkage and reinforcement ratio, it may have a significant influence even in the short-term behavior of RC elements [55,56,57,58]. Considerable shrinkage can be developed in concrete elements before testing unless special attention to the curing method and conditions is paid. With increasing the shrinkage at early age of concrete, tensile stresses are developed in concrete due to the restrain caused by internal reinforcement. This causes a reduction in the cracking moment of the flexural element and a shift on the bare bar response and therefore an increase in deflections.

In this work, shrinkage before flexural loading was assessed since shrinkage strain recorded from the day of casting was significantly higher for specimens in series 2 (344 µε) when compared to that of series 1 (88 µε).

**Figure 7:**
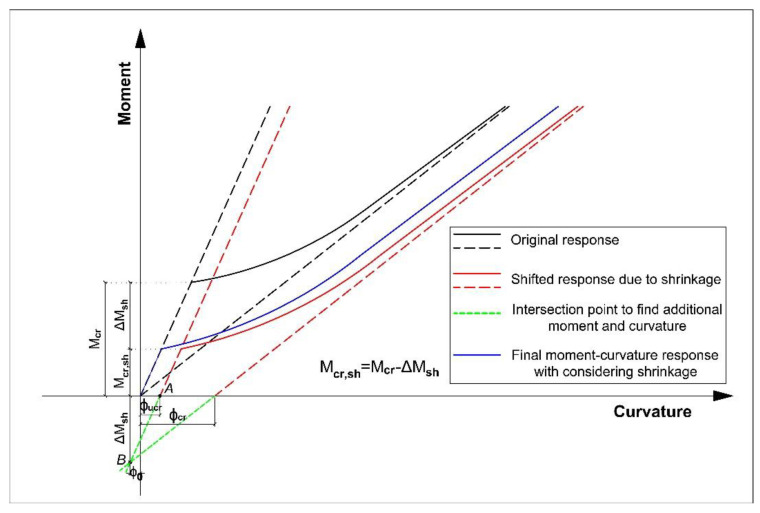
Flexural response of beam with and without shrinkage.

The original uncracked and cracked responses (moment–curvature relationships) of a flexural specimen without considering the shrinkage are shown in Figure 7 (black lines). The corresponding uncracked and cracked additional curvatures due to shrinkage can be calculated according to [59]:

(1) $$\varphi_{ucr}=\frac{A_{ucr}\Delta M_{ucr}+B_{ucr}\Delta N_{ucr}}{E_{c}\left(A_{ucr}I_{ucr}-B_{ucr}^{2}\right)}$$

(2) $$\varphi_{cr}=\frac{A_{cr}\Delta M_{cr}+B_{cr}\Delta N_{cr}}{E_{c}\left(A_{cr}I_{cr}-B_{cr}^{2}\right)}$$

where *E_c_* is the modulus of elasticity of concrete, *A_ucr_* is the area of uncracked transformed section, and *B_ucr_* and *I_ucr_* are the first and second moments of inertia of the area of the uncracked transformed section, respectively. Moreover, *A_cr_*, *B_cr_*, and *I_cr_* are the corresponding values for the cracked transformed section. Furthermore, ∆*M* and ∆*N* are the restraining moment and axial force against shrinkage.

(3) $$\Delta N_{ucr}=-E_{c}\varepsilon_{sh}A_{c,ucr}$$

(4) $$\Delta M_{ucr}=E_{c}\varepsilon_{sh}B_{c,ucr}$$

(5) $$\Delta N_{cr}=-E_{c}\varepsilon_{sh}A_{c,cr}$$

(6) $$\Delta M_{cr}=E_{c}\varepsilon_{sh}B_{c,cr}$$

where *ε_sh_* is the shrinkage in concrete and *A_c_* and *B_c_* are the area of concrete and the first moment of inertia of the area of concrete (ignoring steel as shrinkage takes place only in concrete).

To account for the effect of shrinkage, these uncracked (*φ_ucr_*) and cracked (*φ_cr_*) additional curvatures are applied, so that the black dash lines in Figure 7 are shifted to the right (red dash lines). To find the additional moment due to shrinkage (∆*M_sh_*), the intersection point between red dash lines is obtained (point B). This means that if shrinkage of the flexural element is accounted for, point B should be the origin of the moment–curvature response. Hence, a reduction in the cracking moment equal to ∆*M_sh_* should be applied (moving from black solid line to red solid line in Figure 7). Then, point A should be shifted to the origin of the absolute coordinate system (moving from the red solid line to the blue solid line in Figure 7). Finally, the blue solid line is the final moment–curvature response of the beam when shrinkage effects are considered. It can be observed that if shrinkage is accounted for, the cracking moment reduces from *M_cr_* to *M_cr,sh_* and additional deflections appear due to an extra curvature.

**Figure 8:**
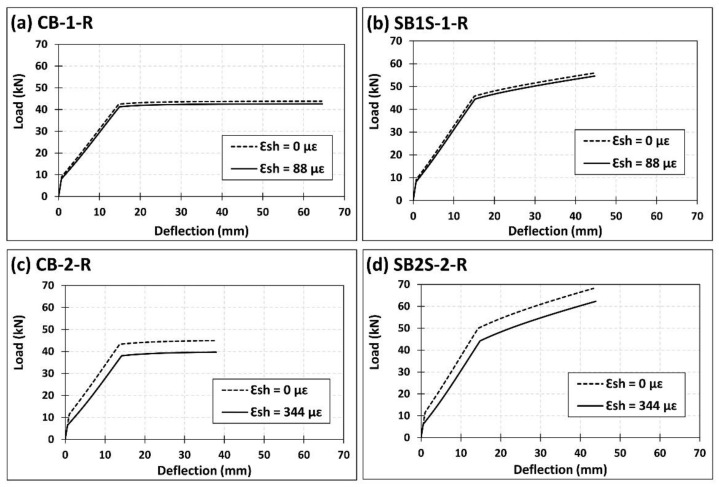
Analytical predictions of load–deflection curves with and without accounting for the shrinkage effect for specimens (**a**) CB-1-R; (**b**) SB1S-1-R; (**c**) CB-2-R; and (**d**) SB2S-2-R.

Representative comparisons between the analytical predictions of load–deflection curves for beams (both unstrengthened and strengthened) of the present work, with and without considering the effect of shrinkage are presented in Figure 8. A larger difference between the two predictions exists for those beams of series 2, as expected.

### 3.2. Load–Deflection Curves

**Figure 9:**
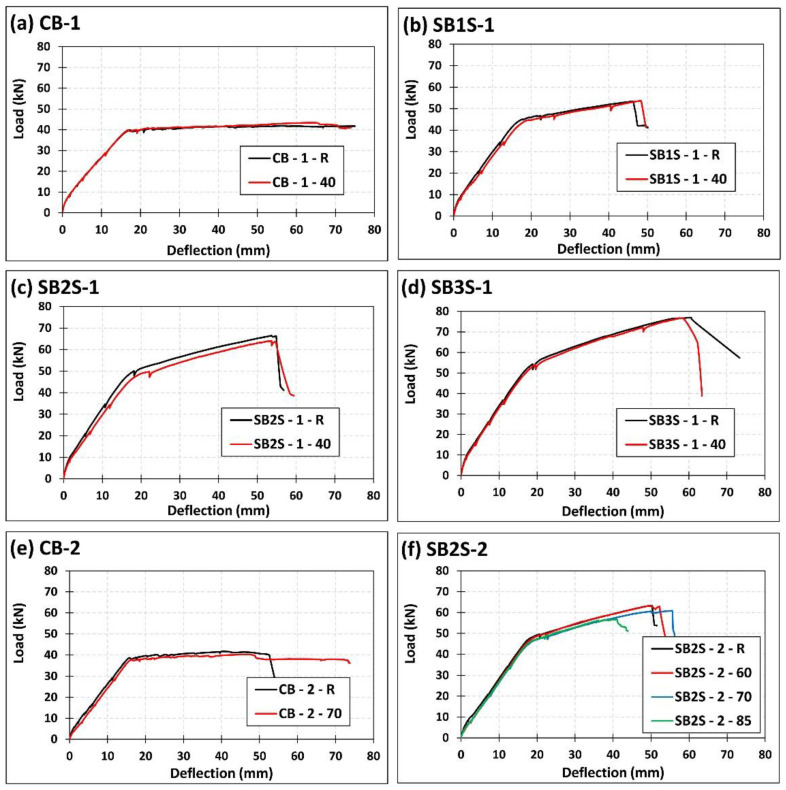
Effect of temperature on the experimental load–deflection curves of specimens (**a**) CB-1; (**b**) SB1S-1; (**c**) SB2S-1; (**d**) SB3S-1; (**e**) CB-2; and (**f**) SB2S-2.

The experimental load versus midspan deflection of the specimens of the present work is presented in Figure 9. As a general description, an initial linear behavior is observed that represents the elastic behavior of the flexural element. Once the cracking load is attained, the stiffness of the system decreases, and the load can be further increased until the yielding load; after this yielding load, the stiffness drops dramatically. From the yielding point onwards, no considerable increase in the ultimate capacity of control RC beams (unstrengthened) is found, whilst the ultimate capacity of strengthened beams is increased. In this sense, with the increase in the strengthening level (i.e., CFRP area), a slight increase of the cracking load was observed (see Table 3). In the strengthened specimens, the stiffness of load–deflection curves was relatively higher than in the RC beams, as expected. Moreover, the yielding (*P_y_*) and ultimate (*P_u_*) loads of the strengthened specimens were accordingly increased, irrespective of the applied temperature (see Figure 9).

**Figure 10:**
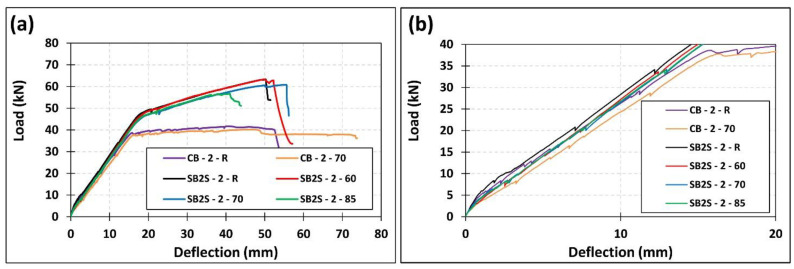
Effect of temperature on the experimental load–deflection curves of series 2 of specimens: (**a**) Total view; (**b**) Zoom in view.

According to Figure 9, small differences can be observed between the load–deflection curves of specimens in series 1 when the temperature was increased from 20 to 40 °C. In this sense, the application of 40 °C derived in a slight reduction of the stiffness in load–deflection curves, which is in accordance with results presented in [32,33,34]. This reduction in stiffness was followed by a slight decrease in the ultimate capacity of the beams. In series 2, the application of a temperature that is approaching and even exceeding the epoxy *T*_g_ (i.e., 60 to 85 °C) generated a small reduction in the stiffness of the specimens. Moreover, the ultimate capacity of the beams in this series decreased by 3.48%, 3.95%, and 10.45% for CB-2-70, SB2S-2-70, and SB2S-2-85, respectively. No change was observed in SB2S-2-60 specimen. In unstrengthened beams, the increase in the temperature derived in a reduction of the cracking load and stiffness of system (see Figure 10). This reduction has been also observed in strengthened beams, where the increase in the temperature (near or beyond *T*_g_) was followed by bond decrease and, subsequently, the efficiency of strengthening system was slightly reduced, thus making beams more susceptible to premature debonding.

Experimental results in terms of cracking load (*P_cr_*), yielding load (*P_y_*), ultimate load (*P_u_*), ultimate CFRP strain (*ε_u,FRP_*), and failure modes are presented and compared to analytical predictions in Table 3. For the analytical predictions, the effect of temperature on normal weight concrete properties is considered following the equations proposed in fib Model Code 2010 [60], which is said to be valid for temperatures ranging approximately from 0 to 80 °C:

(7) $$f_{cm}\left(T\right)=f_{cm}\left(1.06-0.003T\right)$$

(8) $$f_{ct,sp}\left(T\right)=f_{ct,sp}\left(1.06-0.003T\right)$$

(9) $$E_{c}\left(T\right)=E_{c}\left(1.06-0.003T\right)$$

where *f_cm_(T)*, *f_ct,sp_(T*), and *E_c_(T)* are the compressive strength, the splitting tensile strength, and the modulus of elasticity of concrete at temperature *T* (in °C), respectively. Moreover, *f_cm_*, *f_ct,sp_*, and *E_c_* are the compressive strength, the splitting tensile strength, and the modulus of elasticity of concrete at *T* = 20 °C, respectively.

The mechanical properties of steel reinforcement and CFRP strips were not modified to include any effect of temperature because they are not sensitive to this range of temperatures [25,26,31,61]. For CFRP material, the ultimate strain (stress) value obtained from experimental results was used in analytical predictions.

**Figure 11:**
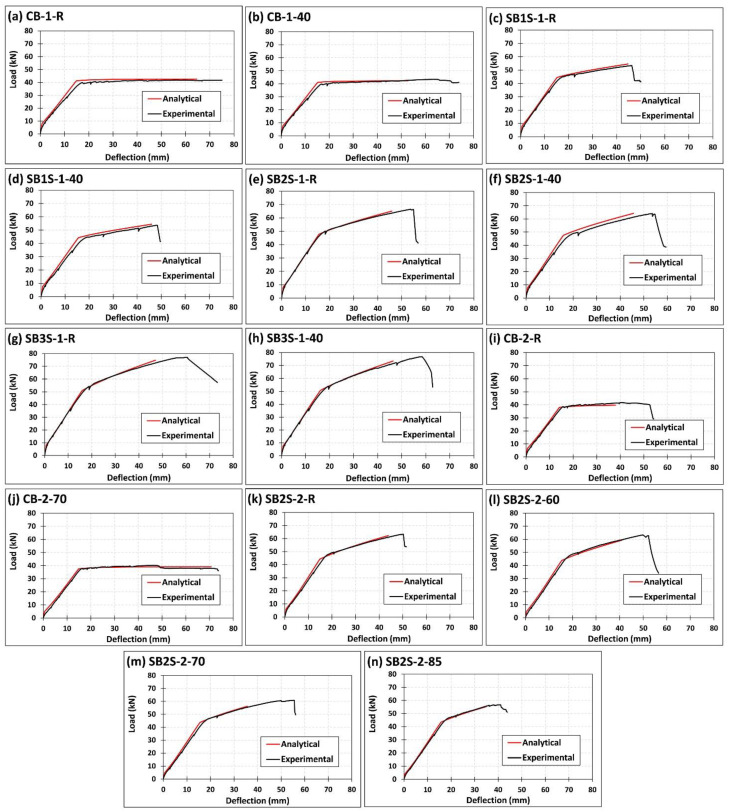
Comparison between experimental and analytical predictions on load–deflection curves of specimens (**a**) CB-1-R; (**b**) CB-1-40; (**c**) SB1S-1-R; (**d**) SB1S-1-40; (**e**) SB2S-1-R; (**f**) SB2S-1-40; (**g**) SB3S-1-R; (**h**) SB3S-1-40; (**i**) CB-2-R; (**j**) CB-2-70; (**k**) SB2S-2-R; (**l**) SB2S-2-60; (**m**) SB2S-2-70; and (**n**) SB2S-2-85.

To complement the comparison between experimental results and analytical predictions presented in Table 3, experimental and analytical load–deflection curves are compared in Figure 11. According to the figure, good agreement between experimental results and analytical predictions can be observed in terms of the cracking load, yielding load (and their corresponding deflections), and stiffness of the system before and after cracking load and after yielding load, thus meaning that shrinkage and temperature were correctly accounted for in the analytical work. However, it should be noted that some differences exist between experimental and analytical ultimate load and deflections.

### 3.3. Failure Mode

**Figure 12:**
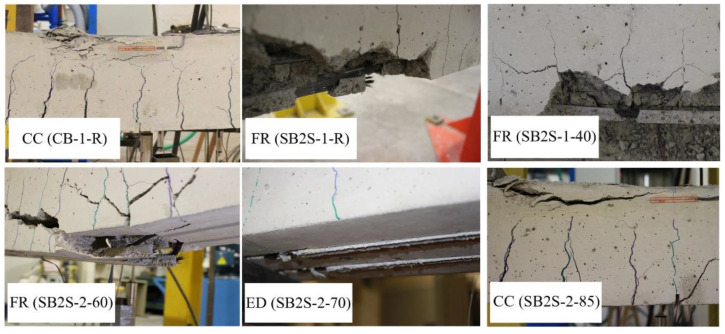
Experimental failure modes.

According to Table 3, control unstrengthened beams of both series (i.e., CB-1 and CB-2, at different temperatures) failed by concrete crushing in the compression zone of the beam after steel yielding. On the other hand, FRP rupture (after yielding of steel reinforcement) was the failure mode of all strengthened beams of series 1. This means that with the application of 40 °C, the anchorage length of the CFRP strips was long enough to avoid FRP debonding failure, and the effect of the application of temperatures within this range is negligible. In series 2 of specimens, the application of 60 °C (i.e., specimen SB2S-2-60) resulted in FRP rupture failure mode, while the application of 70 °C (i.e., specimen SB2S-2-70) lead to failure by CFRP end debonding. To conclude, in specimen SB2S-2-85 (i.e., application of 85 °C), concrete was initially crushed in the compression zone of the section and, as a result, the load suddenly dropped without end debonding. This unexpected behavior can be due to a change in concrete mechanical properties, FRP, and epoxy at high service temperatures. As a general comment regarding the failure mechanism of the specimens, the increase in the temperature may be followed by a reduction of the efficiency of the strengthening system and, subsequently, it can lead to changes in the failure mechanism of the system (changing from FRP rupture to FRP debonding or even concrete crushing). Representative images of experienced failure modes are shown in Figure 12. It should be noted that in those cases where FRP rupture took place, it was subsequently followed by the detachment of the concrete around the FRP laminate.

As previously mentioned, fib Bulletin 90 [8] recommends limiting the maximum temperature to avoid premature failure in EBR-strengthened systems. According to experimental observations, as long as the temperature is sufficiently lower than *T*_g_, no premature failure was observed. On the contrary, the application of a temperature exceeding the *T*_g_ resulted in end debonding failure mode (i.e., specimen SB2S-2-70). It should be noted that no premature failure (i.e., end debonding) occurred when temperature in the range of *T*_g_ was applied (*T* = 60 °C), thus showing a good performance related to end debonding for the NSM strengthening system. Nevertheless, it must be noted that additional experimental work is needed to confirm the findings of the experimental program presented in this communication.

### 3.4. Strain Distribution along the CFRP Strip

**Figure 13:**
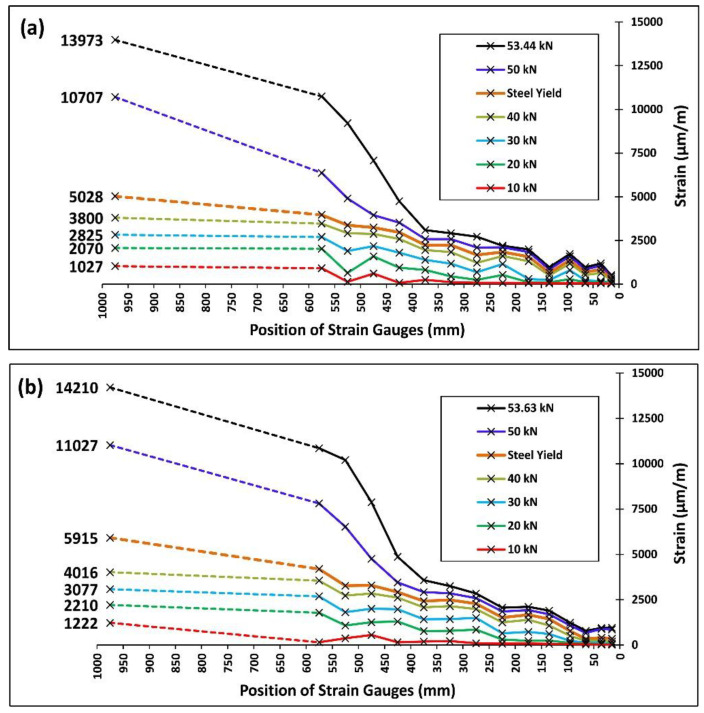
Strain distributions along one half of the CFRP strip at different levels of load for specimens (**a**) SB1S-1-R and (**b**) SB1S-1-40.

The distribution of strains along the CFRP strips can give useful information about the bond behavior (i.e., the force distribution between the CFRP and concrete interface) and its influence on cracking phenomena. Instrumentation with strain gauges presented in Figure 3b allowed the register of strains at several sections along the CFRP strip in SB1S-1-R and SB1S-1-40 specimens. Experimental strain distributions along half of the CFRP laminate of these specimens are plotted at different levels of load in Figure 13. Experimental results show that the increase in temperature was followed by an increase of strains in the strip. In addition, in both specimens, the strain in the CFRP strip increased dramatically after yielding of the longitudinal steel reinforcement. The available fluctuation in the strain distributions may be related to the appearance and opening of new cracks.

## 4. Finite Element Analysis

Nonlinear finite element (FE) analysis of control and CFRP-strengthened beams subjected to four-pointed loads was carried out to compare with the results obtained from the experimental work. A three dimensional FE model was created using the well-known commercial FE program ABAQUS [62].

### 4.1. Description of FE Model

**Figure 14:**
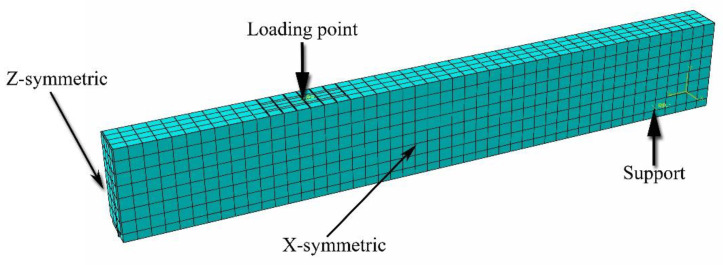
Finite element mesh of simulated beam (one quarter).

FE models include concrete, steel reinforcements (longitudinal and stirrups), CFRP strips, and epoxy. Concrete and epoxy are modeled by an eight-node linear hexahedral solid element with reduced integration (C3D8R), steel reinforcements are modeled by a two-node linear 3D truss element (T3D2), and FRP strips are modeled by a two-node linear beam (B31). A mesh convergence analysis was performed to select the optimum mesh sizes for different parts involved in the FE model. In this study, it was observed that the meshing refinement was not sensitive after having the maximum mesh size of 25 mm for all elements except epoxy that had 10 mm mesh size. By taking advantage of symmetry in two perpendicular planes, only a quarter of the beam was modeled. Boundary conditions of the experimental test set-up were considered. Finite element mesh along with the boundary conditions is shown in Figure 14.

Considering perfect bond, steel reinforcements and CFRP strips are embedded inside the concrete and epoxy, respectively. In order to address this condition, the embedded region function of ABAQUS is used. Moreover, the tie constraint function is assumed to simulate the interaction in the interface between epoxy and concrete. The selection of these interfacial behaviors is based on the fact that the bond was good enough and bond failure took place only in one of the beams of the present experimental program (i.e., specimen SB2S-2-70). As a result of this, specimen SB2S-2-70 is not included in the numerical simulations.

Similar to the case of analytical predictions presented in Section 3.1, the effect of shrinkage is also addressed in the numerical simulations. To this end, an initial extra deflection due to shrinkage (calculated following Section 3.1) is applied to the middle of the beam in a separate step. Afterwards, the main flexural load is applied in a displacement control mode through a rigid plate to avoid any extra deformation along the loading.

### 4.2. Materials Definition

Steel reinforcement is modeled as an isotropic bilinear elasto-plastic material, while epoxy is modeled as isotropic linear elastic material and CFRP is modeled as brittle material (with ultimate strain value obtained from experimental results). Moreover, to simulate the inelastic behavior of concrete, concrete damage plasticity (CDP) is used. This continuum, plasticity-based, damage model for concrete is applicable to different loading conditions and can be utilized for concrete with embedded reinforcement [62]. This model assumes that the two main failure mechanisms are tensile cracking and compressive crushing. Therefore, the evolution of the failure surface is controlled by two hardening variables linked to the failure mechanisms under tension and compression loading. The required material parameters for the definition of the CDP model are dilation angle (*ψ*), flow potential eccentricity (*є*), the ratio of the biaxial compressive stress to the uniaxial compressive stress (*σ_b_*_0_*/σ_c_*_0_), the ratio of the second stress invariant on the tensile meridian to that on the compressive meridian (*K_c_*), and viscosity (*µ*). In this numerical study, these parameters are considered as *ψ* = 36°, *є* = 0.1, *σ_b_*_0_*/σ_c_*_0_ = 1.16, *K_c_* = 0.67, and *µ* = 0.0005.

**Figure 15:**
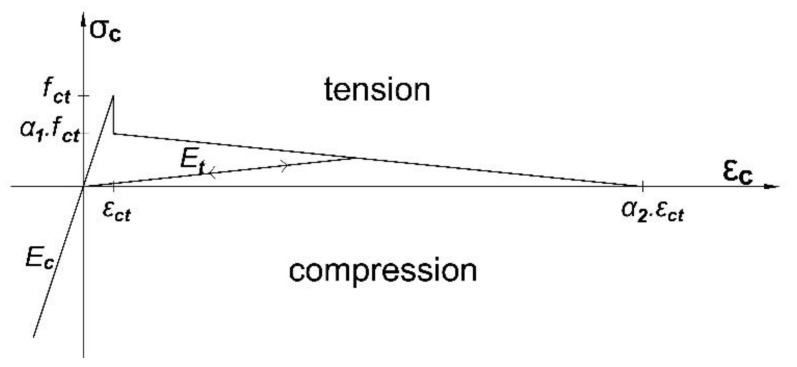
Equivalent stress–strain relationship for tensioned concrete [64].

The concrete stress–strain compression relationship is modeled according to Eurocode 2 [63]. Moreover, the tensile post-cracking behavior of concrete is modeled according to Torres et al. [64], where *α*_1_ and *α*_2_ are two dimensionless coefficients that define the tensile post-cracking stress–strain relationship of concrete (see Figure 15). In this study, based on the loading condition, *α*_1_ was assumed to be equal to 0.4 and based on section properties, *α*_2_ was assumed to be in the range of 13 to 15.

The effect of temperature is applied directly in the definition of materials’ properties, following the same strategy as in the analytical work (see Section 3.2). The effect of temperature on epoxy material is considered according to the experimental results presented in [7]. This assumption is based on the fact that the possible effect of the heating process, taking place before the main flexural test, is not included in the experimental load–deflection responses obtained in the laboratory (i.e., the system is zeroed before testing).

### 4.3. FE Results

**Figure 16:**
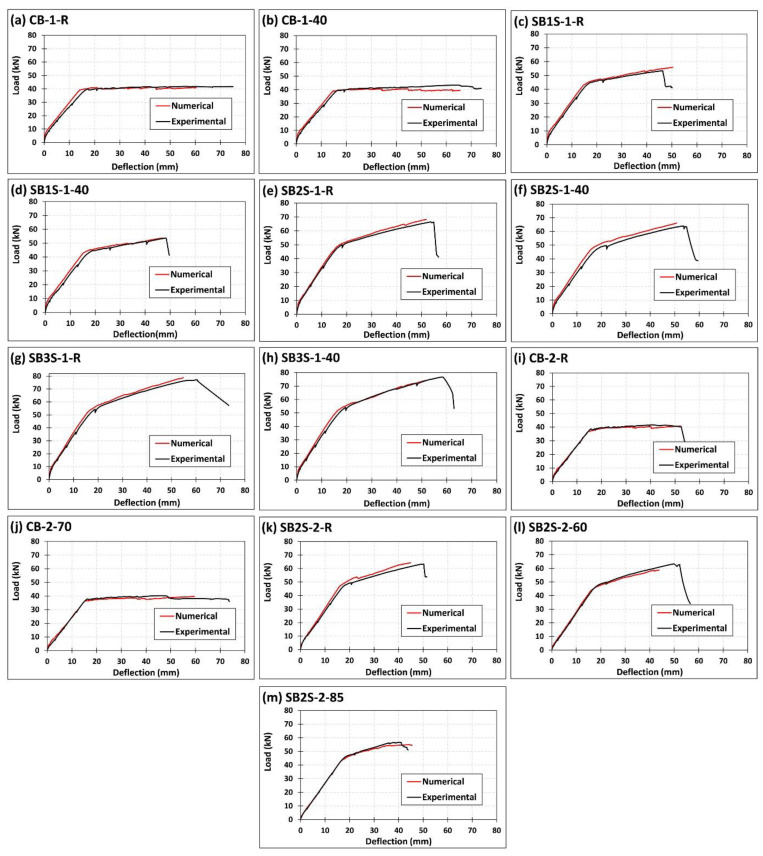
Comparison between experimental and numerical work of specimens (**a**) CB-1-R; (**b**) CB-1-40; (**c**) SB1S-1-R; (**d**) SB1S-1-40; (**e**) SB2S-1-R; (**f**) SB2S-1-40; (**g**) SB3S-1-R; (**h**) SB3S-1-40; (**i**) CB-2-R; (**j**) CB-2-70; (**k**) SB2S-2-R; (**l**) SB2S-2-60; and (**m**) SB2S-2-85.

The comparison between numerical predictions and experimental results is presented in Figure 16 and Table 4. In all cases, the FE model reasonably predicts the experimental behavior of beams under flexural load. Slightly higher initial stiffness is observed in the numerical simulation when compared to experimental curves, with a mean difference in deflections of 7.15% observed at the service load level [8]. In terms of yielding and ultimate loads, differences of only 3% and 2.9% are observed, respectively, with the same failure mode in both experimental work and numerical simulations. In this sense, it should be highlighted that concrete crushing was the predicted failure mode in the numerical simulation of specimen SB2S-2-85, which coincides with experimental observation. This would confirm that the negative effect of temperature on the mechanical properties of concrete and epoxy leads to an unexpected concrete crushing failure mode.

## 5. Conclusions

In the present work, an experimental program to study the influence of high service temperature on the flexural performance of NSM CFRP-strengthened RC beams is presented. The experimental results are compared to analytical and numerical predictions. Based on the data presented, the following conclusions can be drawn:

- No considerable differences can be observed in the load–deflection curve when specimens are subjected to 40 °C (i.e., series 1). This behavior can be attributed to 40 °C being far below the *T*_g_ of the epoxy resin.
- In series 2, as a result of the increase in temperature near or beyond the *T*_g_, the stiffness and the ultimate load of strengthened and unstrengthened specimens decreased. The interface bond capacity between epoxy and concrete could have been affected, and the efficiency of the strengthening system showed some reduction, thus making the beam to be more prone to experience end deboning failure.
- In both series of specimens, the failure mode of the control unstrengthened beams was concrete crushing after steel yielding. For the strengthened beams under 20, 40, and 60 °C, the failure mode was FRP rupture. With the increase of temperature up to 70 °C, the beam failed by end debonding. Finally, specimens under 85 °C experienced concrete crushing and, as a result, the load suddenly dropped without giving place to end debonding. This failure mode can be attributed to the reduction in mechanical properties of concrete and epoxy when submitted to higher temperature.
- No end debonding or significant reduction in ultimate load occurred for specimens being tested up to 60 °C, thus showing a good performance of the NSM strengthening system.
- The application of temperature exceeding the epoxy *T*_g_ generated a small reduction in the stiffness of the specimen and a decrease by 3.48%, 3.95%, and 10.45% in the ultimate capacity of beams CB-2-70, SB2S-2-70, and SB2S-2-85, respectively.
- The effect of temperature on the experimental bond behavior of specimen at 40 °C was studied in one of the strengthened beams (i.e., SB1S-1 specimens). With the increase in the temperature, strains (and stresses) in the CFRP strip increased.
- A systematic procedure was applied to consider the effect of shrinkage in short-term response of the specimens. It was observed that the increase in the concrete shrinkage resulted in a reduction of the elements’ stiffness and cracking load. Good agreement was observed between analytical predictions and experimental results.
- A simple but trustable FE model accounting for initial shrinkage and effects of temperature was created to be compared with experimental work. In all cases, numerical models correctly predict the experimental load–deflection responses and failure modes.

## Figures and Tables

**Table 1:** Details of the tested specimens.

Experimental Campaign	Beam ID	Test Temperature (°C)	Concrete Compressive Strength (MPa)	FRP Dimension *w* × *h* (mm^2^)	No. of Strips	CFRP Area (mm^2^)
Series 1	CB-1-R	20	31.8	-	-	-
	CB-1-40	40		-	-	-
	SB1S-1-R	20		1.4 × 10	1	14
	SB1S-1-40	40		1.4 × 10	1	14
	SB2S-1-R	20		1.4 × 10	2	28
	SB2S-1-40	40		1.4 × 10	2	28
	SB3S-1-R	20		1.4 × 10	3	42
	SB3S-1-40	40		1.4 × 10	3	42
Series 2	CB-2-R	20	40.8	-	-	-
	CB-2-70	70		-	-	-
	SB2S-2-R	20		1.4 × 10	2	28
	SB2S-2-60	60		1.4 × 10	2	28
	SB2S-2-70	70		1.4 × 10	2	28
	SB2S-2-85	85		1.4 × 10	2	28

**Table 2:** Results of DSC and DMA methods.

Methodology	DSC			DMA		
	*T*_f_	*T*_m_	*T*_e_	Storage Modulus (*E′*)	Loss Modulus (*E″*)	Loss Factor (*tanδ*)
*T*_g_ (°C)	53.9	55.2	55.9	56.2	57.7	65.3

**Table 3:** Experimental results and analytical predictions.

Beam ID	Cracking Load, *P_cr_* (kN)		Yielding Load, *P_y_* (kN)		Ultimate Load, *P_u_* (kN)		Ultimate CFRP Strain, *ε_u, FRP_* (mm/mm)	Failure Mode ^1^	
	Exp.	Analyt.	Exp.	Analyt.	Exp.	Analyt.	Exp.	Exp.	Analyt.
CB-1-R	6.53	7.98	39.90	41.29	41.87	42.59	-	CC	CC
CB-1-40	5.51	7.48	39.68	41.07	43.39	42.23	-	CC	CC
SB1S-1-R	7.11	7.97	44.90	44.68	53.44	54.57	0.0139	FR	FR
SB1S-1-40	6.07	7.47	44.69	44.36	53.63	54.39	0.0142	FR	FR
SB2S-1-R	7.21	7.96	49.40	47.90	66.50	65.04	0.0132	FR	FR
SB2S-1-40	6.12	7.46	49.27	47.68	64.06	64.19	0.0134	FR	FR
SB3S-1-R	7.56	7.95	56.80	51.29	77.04	74.74	0.0127	FR	FR
SB3S-1-40	6.41	7.45	55.60	51.03	76.77	73.39	0.0124	FR	FR
CB-2-R	3.69	6.17	38.59	38.12	41.71	39.72	-	CC	CC
CB-2-70	2.12	4.66	37.76	37.64	40.26	39.14	-	CC	CC
SB2S-2-R	4.79	5.67	48.21	44.51	63.36	62.27	0.0131	FR	FR
SB2S-2-60	2.09	4.45	47.88	43.99	63.39	59.35	0.0119	FR	FR
SB2S-2-70	1.82	4.15	46.70	43.80	60.86	56.21	0.0103	ED	FR
SB2S-2-85	1.68	3.69	46.47	43.70	56.72	55.24	- ^2^	CC	FR

^1^ Failure modes. CC: concrete crushing after steel yielding; FR: FRP rupture; ED: end debonding; ^2^ Strain gauge failed during the loading.

**Table 4:** Comparison between numerical predictions and experimental results.

Beam ID	Service Load (kN)	Deflection at Service Load, *d_s_* (mm)		Diff. (%)	Yielding Load, *P_y_* (kN)		Diff. (%)	Ultimate Load, *P_u_* (kN)		Diff. (%)	Failure Mode ^1^	
		Exp.	Numeric.		Exp.	Numeric.		Exp.	Numeric.		Exp.	Numeric.
CB-1-R	20.57	7.04	5.99	14.91	39.90	39.15	1.88	41.87	41.22	1.55	CC	CC
CB-1-40	19.82	6.65	6.07	8.72	39.68	39.11	1.44	43.39	40.75	6.08	CC	CC
SB1S-1-R	21.37	6.70	5.93	11.49	44.90	43.39	3.36	53.44	55.86	4.53	FR	FR
SB1S-1-40	20.59	7.01	5.94	15.26	44.69	43.18	3.38	53.63	53.73	0.19	FR	FR
SB2S-1-R	22.12	6.10	5.82	4.59	49.40	49.99	1.19	66.50	68.20	2.56	FR	FR
SB2S-1-40	21.32	6.60	5.78	12.42	49.27	48.83	0.89	64.06	66.07	3.14	FR	FR
SB3S-1-R	22.85	6.02	5.67	5.81	56.80	54.11	4.74	77.04	78.77	2.25	FR	FR
SB3S-1-40	22.01	5.97	5.51	7.71	55.60	53.76	3.31	76.77	74.38	3.11	FR	FR
CB-2-R	26.28	9.98	9.99	0.10	38.59	36.95	4.25	41.71	40.78	2.23	CC	CC
CB-2-70	24.64	10.20	10.05	1.47	37.76	36.38	3.65	40.26	39.60	1.64	CC	CC
SB2S-2-R	29.20	10.31	9.55	7.37	48.21	47.49	1.49	63.36	64.24	1.39	FR	FR
SB2S-2-60	27.04	9.94	9.65	2.92	47.88	45.71	4.53	63.39	58.72	7.37	FR	FR
SB2S-2-85	25.63	9.52	9.51	0.11	46.47	44.34	4.58	56.72	55.55	2.06	CC	CC
		Mean diff. (%)		7.15	Mean diff. (%)		2.98	Mean diff. (%)		2.93		

^1^ Failure modes. CC: concrete crushing after steel yielding; FR: FRP rupture.

## Data Availability

Not applicable.
